# A Systematic Review and Meta-Analysis of Prophylactic Anticoagulation for the Prevention of Catheter-Related Thrombosis in Adult Cancer Patients with Long-Term Central Venous Catheters: Current Evidence, Clinical Uncertainties and Future Directions

**DOI:** 10.3390/jcm15145566

**Published:** 2026-07-15

**Authors:** Jagoda Kania, Jacek Zawadzki, Bartosz Kudliński

**Affiliations:** Department of Anaesthesiology, Intensive Care and Emergency Medicine, University of Zielona Góra, 65-046 Zielona Góra, Poland; jacekzawadzkimed@gmail.com (J.Z.); bartoszkudlinski@gmail.com (B.K.)

**Keywords:** cancer, long-term central venous catheter, thromboprophylaxis, catheter-related thrombosis, major bleeding

## Abstract

**Objectives:** Cancer patients frequently require long-term central venous catheters to facilitate chemotherapy administration. However, catheter-related thrombosis represents a clinically relevant complication that may interrupt cancer treatment. The role of pharmacological thromboprophylaxis in preventing catheter-related thrombosis remains controversial due to concerns regarding bleeding risk. This systematic review and meta-analysis aimed to evaluate the efficacy and safety of prophylactic anticoagulation for the prevention of catheter-related thrombosis in adult cancer patients with long-term central venous catheters. **Methods:** A systematic search of PubMed, Cochrane Library, Web of Science, Scopus, and ClinicalTrials.gov was conducted. Randomized controlled trials and observational studies evaluating prophylactic anticoagulation in adult cancer patients with long-term central venous catheters were included. The primary outcomes were catheter-related thrombosis and major bleeding. Risk of bias was assessed using RoB 2 and ROBINS-I. Meta-analyses were performed using random-effects models where feasible. **Results:** Sixteen studies were included in this systematic review. Separate quantitative syntheses were performed for randomized and non-randomized studies. In the meta-analysis of three randomized controlled trials including 1022 patients, prophylactic low-molecular-weight heparin did not significantly reduce the risk of catheter-related thrombosis compared with control (RR 0.77; 95% CI 0.53–1.12). A separate analysis of two non-randomized studies including 593 patients suggested a lower reported incidence of catheter-related thrombosis with rivaroxaban (RR 0.23; 95% CI 0.11–0.48). Major bleeding outcomes were inconsistently reported across studies and could not be quantitatively synthesized. **Conclusions:** Current evidence does not support routine prophylactic anticoagulation for all cancer patients with long-term central venous catheters. However, selected high-risk patients may potentially benefit from individualized thromboprophylaxis. The available evidence remains limited by heterogeneity, low certainty and inconsistent reporting of bleeding outcomes. Further adequately powered randomized trials are required.

## 1. Introduction

Patients with cancer face multiple challenges related to oncological treatment. The administration of chemotherapy often requires the use of specialized central venous catheters (CVCs), which enable safe and prolonged intravenous access while minimizing complications such as phlebitis or extravasation.

Patients with cancer are at an increased risk of thrombotic events due to malignancy-associated hypercoagulability, cancer-directed therapies, and frequent exposure to invasive procedures. The presence of a central venous catheter further augments this risk through endothelial injury and venous stasis.

One of the most clinically significant complications affecting both the course of treatment and patients’ quality of life is catheter-related venous thrombosis. Catheter-related thrombosis (CRT) is one of the most common complications associated with long-term central venous catheters in patients with cancer. Reported incidence ranges from approximately 2% to over 18%, depending on catheter type, tumour characteristics, and diagnostic strategy. Importantly, reported incidence is also influenced by whether symptomatic or asymptomatic thrombosis is considered [1,2,3]. Although many thrombotic events remain clinically silent, CRT may result in catheter dysfunction, the interruption of anticancer treatment, need for catheter removal, therapeutic anticoagulation, pulmonary embolism and increased healthcare utilization [4], but the contemporary magnitude of these clinically relevant consequences of CRT is unknown. Similar observations have been reported for cancer-associated venous thromboembolism, where incidental and symptomatic thromboembolic events differ in their clinical presentation and prognosis, highlighting the importance of distinguishing clinically relevant outcomes [5]. Consequently, the role of prophylactic anticoagulation in cancer patients with long-term central venous catheters warrants careful evaluation, particularly in the context of potential bleeding complications associated with anticoagulant therapy. Previous studies and systematic reviews have suggested a potential benefit of pharmacological thromboprophylaxis; however, heterogeneity in study design, anticoagulant type, and outcome definitions has limited the certainty of these findings [6,7]. Accordingly, current guidelines do not recommend standardized thromboprophylaxis in cancer patients receiving central venous catheters [8,9,10,11,12,13]. Previous meta-analyses largely focused on low-molecular-weight heparin (LMWH) and pooled heterogeneous anticoagulant strategies together. Moreover, contemporary data regarding direct oral anticoagulants and their potential role in preventing catheter-related thrombosis remain limited.

Given substantial advances in catheter materials, insertion techniques, and thrombosis prevention strategies over the past two decades, the contemporary interpretation of available evidence requires the careful consideration of temporal changes in clinical practice. Despite the growing body of literature, reported estimates vary considerably across studies because of differences in catheter types, study populations, outcome definitions and diagnostic approaches, making the interpretation of available evidence challenging.

Therefore, we conducted a systematic review and meta-analysis to evaluate the efficacy and safety of pharmacological thromboprophylaxis for the prevention of catheter-related thrombosis in adult cancer patients with long-term central venous catheters.

## 2. Materials and Methods

### 2.1. Study Design and Protocol Registration

This systematic review and meta-analysis was conducted in accordance with the PRISMA 2020 statement [14]. The completed PRISMA 2020 checklist is provided in the Supplementary Materials as Table S1. The study protocol was prospectively registered in the PROSPERO database (registration number: CRD420251249192).

### 2.2. Eligibility Criteria

We included studies that met the following criteria:

1. Adult cancer patients (≥18 years) with implanted long-term central venous catheters.
2. The use of pharmacological thromboprophylaxis (including low-molecular-weight heparin [LMWH], direct oral anticoagulants [DOACs], or warfarin).
3. Randomized controlled trials (RCTs) and observational prospective or retrospective studies.
4. Reported outcomes including catheter-related thrombosis and/or major bleeding.

Studies were excluded if they:

1. Involved the pediatric population.
2. Evaluated therapeutic anticoagulation or anticoagulation for indications other than thromboprophylaxis.
3. Were case reports, case series, reviews, editorials, or non-original research articles.

### 2.3. Outcomes

The primary efficacy outcome was catheter-related thrombosis, including both symptomatic and asymptomatic events as reported in the original studies. Asymptomatic CRT was primarily diagnosed by scheduled ultrasonography or venography according to individual study protocols. The primary safety outcome was major bleeding, defined according to the International Society on Thrombosis and Haemostasis (ISTH) criteria whenever available [15].

### 2.4. Search Strategy

A comprehensive literature search was conducted in four electronic databases, PubMed, Cochrane Library, Web of Science, and Scopus, as well as one clinical trial registry (ClinicalTrials.gov). The search was performed up to 7 December 2025.

For each database, a tailored search strategy was developed using combinations of keywords and controlled vocabulary terms (MeSH or equivalents), including: cancer, oncolog*, neoplasms, central venous catheter, CVC, totally implantable venous access device, TIVAD, portacath, PICC, anticoagul*, thromboprophylaxis, prophylaxis, catheter-related thrombosis, CRT, CRVT, central line thrombosis, thrombosis, major bleeding, bleeding, and hemorrhag*.

The full search strategies are provided in the Supplementary Materials, Files S1–S4.

### 2.5. Study Selection and Data Extraction

Two reviewers independently screened titles and abstracts according to the predefined inclusion and exclusion criteria. Full-text articles were subsequently assessed independently by the same reviewers. Any disagreements were resolved through discussion with a third reviewer.

When necessary, additional information was obtained from Supplementary Materials or requested directly from study authors. Extracted data included study characteristics, patient population, type of anticoagulant, comparator, follow-up duration, and reported outcomes.

### 2.6. Risk-of-Bias Assessment

Risk of bias was independently assessed by two reviewers, with discrepancies resolved by a third reviewer. The Cochrane Risk of Bias 2 (RoB 2) [16] tool was used for randomized controlled trials, and the Risk Of Bias In Non-randomized Studies of Interventions (ROBINS-I) [17] tool was applied to non-randomized studies of interventions. Visual summaries of risk-of-bias assessments were generated using the *robvis* tool [18].

### 2.7. Data Synthesis and Statistical Analysis

Meta-analyses were performed using Review Manager (RevMan) [19] software version 7.2.0, The Cochrane Collaboration 2024. Randomized and non-randomized studies were synthesized separately to account for differences in study design and potential risk of bias. Risk ratios (RRs) with 95% confidence intervals were used as the summary measure because all included studies reported dichotomous outcomes. Random-effects models with the Mantel–Haenszel method were applied to calculate pooled risk ratios (RRs) with 95% confidence intervals (CIs). The summary effect was estimated using the Wald-type method.

Statistical heterogeneity was assessed using the DerSimonian and Laird estimator and quantified with the I^2^ statistic. The exploration of sources of heterogeneity was limited due to the small number of included studies; however, subgroup analyses were conducted separately for LMWH and rivaroxaban. These subgroup analyses were predefined based on the anticoagulant used. Sensitivity analyses using fixed-effect models were performed to assess the robustness of the pooled estimates. Forest plots were generated to present the results of meta-analyses.

Publication bias was not formally assessed because fewer than ten studies were available for each meta-analysis, consistent with current methodological recommendations. Overall pooled estimates were generated only when studies were considered sufficiently clinically and methodologically comparable.

### 2.8. Certainty of Evidence

The certainty of evidence for each outcome was assessed by two reviewers using the GRADE [20] approach. Any disagreements were resolved by consensus after discussion with a third reviewer.

## 3. Results

**Figure 1:**
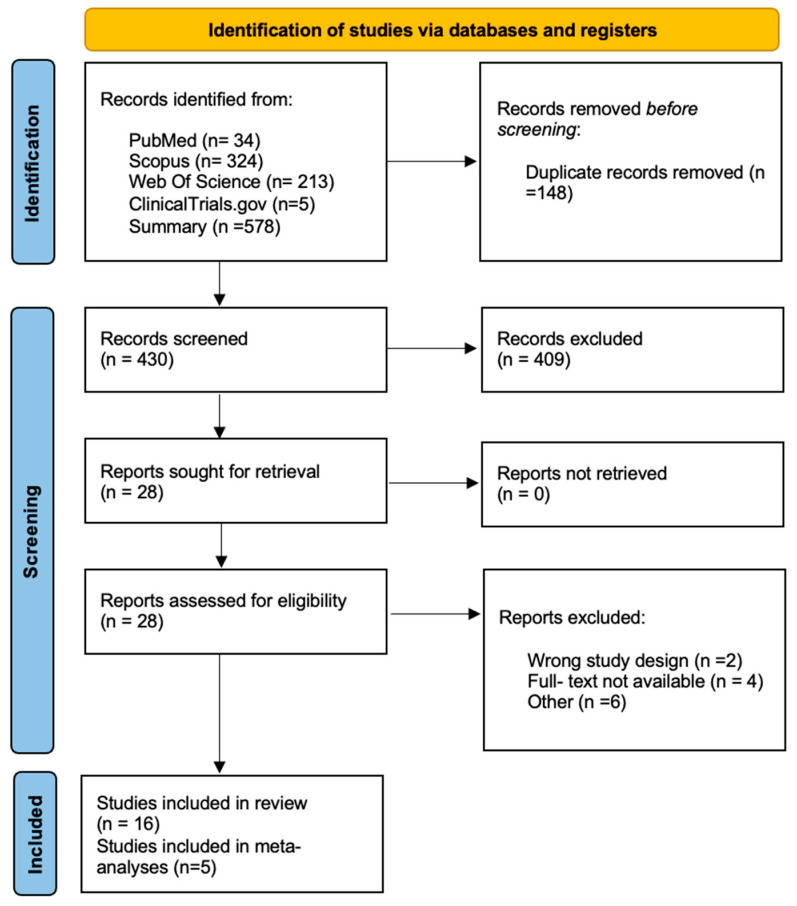
PRISMA flow diagram of study selection [14].

The literature search identified 578 results. After the removal of duplicates, 430 records were screened by title and abstract. A total of 28 full-text articles were assessed for eligibility, of which 12 were excluded. Finally, 16 studies were included in the systematic review, including 8 randomized controlled studies and 8 non-randomized studies [21,22,23,24,25,26,27,28,29,30,31,32,33,34,35,36]. Quantitative synthesis was performed for LMWH and rivaroxaban separately. The search process is summarized in Figure 1. The most common reasons for exclusion were inappropriate study design, non-eligible population and limited possibilities for full-text retrieval. Excluded studies [37,38,39,40,41,42,43,44,45,46] and reasons for exclusion are available in the Supplementary Materials in Table S2.

### 3.1. Study Characteristics and Risk of Bias

The characteristics of included studies are summarized in Table 1. Studies were published between 1998 and 2025 and included adult cancer patients receiving long-term central venous catheters. Interventions included prophylactic-dose anticoagulants initiated at or shortly after the time of CVC implantation. Follow-up ranged from 42 days from implantation up to 6 months after device removal.

Overall risk of bias varied across studies. Most RCTs were judged to have a low risk of bias or some concerns, whereas non-randomized studies were mainly at moderate risk, but some of them were at serious risk of bias, primarily due to confounding. Summarized risk-of-bias assessment is available in the Supplementary Materials as Figures S1–S4.

### 3.2. Results of Individual Studies

The anticoagulants analyzed were warfarin, coumarin, LMWH, rivaroxaban and apixaban, all in prophylactic doses. Individual studies reported a lower incidence of catheter-related thrombosis or a lack of prophylactic effect in patients receiving anticoagulation compared with control groups. The absolute rates of CRT varied between studies, reflecting differences in patient populations, anticoagulant regimens and durations of follow-up.

Major bleeding was reported in seven studies (Table 2). Three studies applied standardized definitions of major bleeding, including ISTH criteria, whereas the remaining studies used study-specific definitions or did not clearly define bleeding outcomes. Across the included studies, no consistent increase in major bleeding associated with thromboprophylaxis was observed. Studies evaluating LMWH reported fewer major bleeding events compared with studies investigating other anticoagulants; however, these findings were inconsistent and based on limited data. Owing to substantial heterogeneity in bleeding definitions, outcome reporting and limited numerical data, quantitative synthesis was not considered appropriate.

### 3.3. Quantitative Synthesis

Five studies were deemed sufficiently homogeneous and were included in quantitative synthesis. Eleven studies were excluded from quantitative synthesis [21,22,23,26,28,29,30,33,34,35,36]. The reasons for exclusion included insufficient or non-comparable outcome data, a lack of appropriate control groups, and study designs not suitable for quantitative pooling. These studies were therefore included in qualitative synthesis only. The precise explanation for study exclusion is available in Table S3.

#### 3.3.1. LMWH vs. Control—Catheter-Related Thrombosis

**Figure 2:**
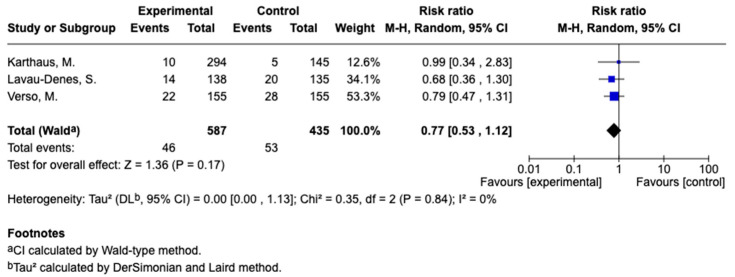
Forest plot of LMWH vs. control for catheter-related thrombosis, including the studies by Karthaus et al. [25], Lavau-Denes et al. [24] and Verso et al. [27].

Three randomized controlled trials were included in the meta-analysis comparing LMWH with control for catheter-related thrombosis (Figure 2). The pooled analysis suggested a possible reduction in the risk of catheter-related thrombosis with LMWH; however, the effect did not reach statistical significance (0.77 RR, 95% CI 0.53, 1.12).

Statistical heterogeneity was not detected (I^2^ = 0%; tau^2^ = 0.00). However, heterogeneity could not be reliably assessed due to the limited number of included studies. Sensitivity analysis using a fixed-effect model did not materially change the results.

#### 3.3.2. Rivaroxaban vs. Control—Catheter-Related Thrombosis

**Figure 3:**
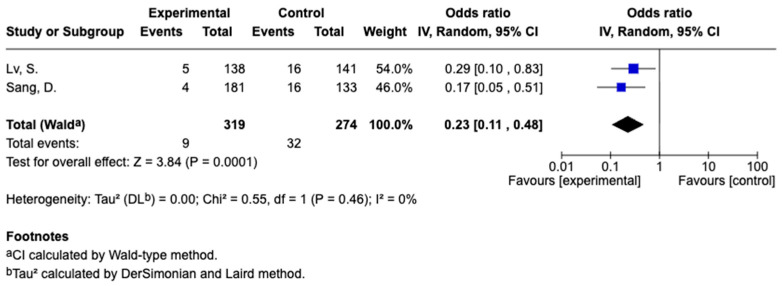
Forest plot of rivaroxaban vs. control for PICC-related thrombosis, including the studies by Lv et al. [31] and Sang et al. [32].

Two studies were included in the meta-analysis evaluating rivaroxaban vs. control (Figure 3). Rivaroxaban use was associated with a lower incidence of PICC-related thrombosis compared with control (RR 0.23, 95% CI 0.11, 0.48).

Given the small number of randomized controlled trials, the precision of the pooled estimate was limited, and a formal investigation of heterogeneity was performed, but heterogeneity was not detected and could not be reliably assessed. Sensitivity analysis using a fixed-effect model, again, did not materially change the results.

Because fewer than ten studies were available for each comparison, formal assessment for publication bias using a funnel plot was not performed.

### 3.4. Certainty of Evidence

The certainty of evidence was rated as low for LMWH and very low for rivaroxaban. A summary of the findings is presented in Table 3.

## 4. Discussion

In the present meta-analysis, prophylactic LMWH showed a consistent direction of effect toward catheter-related thrombosis; however, the pooled estimate did not reach statistical significance. Previous systematic reviews have suggested a potential benefit of pharmacological thromboprophylaxis in reducing catheter-related thrombosis among cancer patients with central venous catheters. Since the publication of these reviews, additional randomized and observational studies evaluating direct oral anticoagulants have become available, warranting an updated synthesis of the current evidence.

Our findings are broadly consistent with those reported by Li at al. [47], who demonstrated that thromboprophylaxis reduced VTE without increasing major bleeding. However, our review extends these findings by incorporating recently published studies, separating randomized and observational evidence and providing an updated assessment focused specifically on catheter-related thrombosis, contemporary anticoagulants and a separate synthesis of randomized and observational evidence.

Similarly, the Cochrane review by Kahale et al. [6] reported a protective effect of LMWH against symptomatic catheter-related thrombosis while emphasizing the low certainty of evidence and the paucity of contemporary randomized data. No clear benefit was observed for asymptomatic thrombosis, and bleeding outcomes favoured no prophylaxis.

From a pathophysiological perspective, the observed reduction in catheter-related thrombosis with pharmacological thromboprophylaxis appears biologically plausible. The presence of central venous catheter fulfils all three components of Virchow’s triad: endothelial injury caused by catheter insertion and chronic contact with the vessel wall, venous stasis and altered flow dynamics due to partial luminal obstruction and flow turbulence, and a hypercoagulable state inherent to malignancy and cancer-directed therapies. Together, these factors provide a thrombotic milieu at the catheter–vessel interface, which may be particularly susceptible to modulation by anticoagulant agents. In this context, prophylactic anticoagulation may attenuate thrombus formation by counteracting the coagulation activation triggered by endothelial disruption and abnormal venous flow [48,49,50,51].

These findings should be interpreted with caution, as the pooled estimates were based on a limited number of studies and did not demonstrate a statistically significant reduction in catheter-related thrombosis with LMWH. The present analysis modestly extends the existing literature by providing a separate synthesis of studies evaluating direct oral anticoagulants, which had not been analyzed independently in prior meta-analyses. The results of our analysis remain largely consistent with the findings of Li et al. and Kahale et al. From a clinical perspective, these findings suggest a possible signal toward benefit with LMWH in selected high-risk patients, although the available evidence remains insufficient to support routine use. Nevertheless, selected patients with a high thrombotic risk profile may derive greater benefit from thromboprophylaxis than unselected cancer populations, supporting an individualized rather than universal preventive strategy. Importantly, cancer-associated hypercoagulability is influenced by tumour type, disease stage, systemic inflammation and concomitant therapies. At the same time, these factors may impair vascular integrity, platelet function and coagulation balance, thereby increasing bleeding susceptibility. Bleeding outcomes were reported inconsistently across the included studies and therefore could not be synthesized quantitatively. Nevertheless, the available evidence did not demonstrate a clear increase in major bleeding associated with thromboprophylaxis, which is broadly consistent with previous systematic reviews, including Li et al. However, less severe bleeding events were reported more frequently in several studies, indicating that the overall safety profile should be interpreted cautiously, particularly in patients with an increased baseline bleeding risk. This dual and dynamic modulation of hemostasis likely explains why the biological rationale for thromboprophylaxis does not consistently translate into clear net clinical benefit across unselected populations.

Routine prophylactic anticoagulation therefore remains controversial, as it is associated with an increased risk of bleeding—an especially important concern in oncological populations who frequently present with thrombocytopenia, mucosal involvement, or treatment-related coagulopathies. Accordingly, current international guidelines do not recommend routine pharmacological thromboprophylaxis solely for the prevention of catheter-related thrombosis, reflecting persistent uncertainty regarding the balance between thrombotic benefit and bleeding risk. Guidelines from ASH, ITAC, and ASCO [8,9,10,11,12,13] emphasize the balance between thrombosis prevention and bleeding risk, highlighting insufficient high-certainty evidence to support universal prophylaxis. The cautious stance adopted by international guidelines may therefore reflect not only the limited statistical power of available trials but also the inherent complexity of thrombotic and bleeding risk stratification in oncology patients. The heterogeneity of the cancer population, catheter types and anticancer treatments complicates the extrapolation of pooled estimates to individual patients. An important source of clinical heterogeneity was the type of central venous device. In particular, the two studies included in the rivaroxaban meta-analysis evaluated patients with peripherally inserted central catheters (PICCs), whereas the LMWH trials included broader long-term central venous catheter populations. Because PICCs are associated with a distinct thrombotic risk profile compared with implantable ports and other long-term central venous devices, the rivaroxaban findings should be interpreted specifically in the context of PICC-related thrombosis rather than generalized to all long-term central venous catheters. This device-related heterogeneity was one reason why an overall pooled estimate across all anticoagulant strategies and catheter types was not considered clinically appropriate [52,53,54].

Importantly, the limited and heterogeneous evidence base underscores that the clinical relevance of thromboprophylaxis may be the greatest in selected subgroups rather than in unselected cancer populations. The net clinical benefit of routine thromboprophylaxis in this setting remains uncertain, appears to vary according to cancer type, comorbidities, patient age, and treatment-related factors, underscoring the need for individualized risk assessment rather than universal prophylactic strategies [55]. Given the inconsistent and sparse reporting of bleeding outcomes, a reliable assessment of net clinical benefit remains challenging. While a reduction in catheter-related thrombosis may be clinically meaningful in patients with high thrombotic burden or limited venous access options, even a small increase in bleeding risk may offset this benefit in patients with thrombocytopenia, mucosal tumours, or intensive chemotherapy regimens.

Consequently, the absence of clearly increased bleeding rates in published studies should not be interpreted as definitive evidence of safety, particularly given the inconsistent reporting of hemorrhagic outcomes.

Advances in cancer-associated thrombosis management resulted in the application of risk assessment models to identify cancer patients with high VTE risk advising medical thromboprophylaxis [56], but the pharmacological prevention of catheter-related thrombosis remains an unresolved area of supportive oncology care. Current recommendations are largely based on low-certainty evidence generated from unselected populations of cancer patients, while contemporary data regarding direct oral anticoagulants, modern catheter techniques, and individualized risk stratification remain scarce [57,58,59,60].

Future research should move beyond evaluating whether thromboprophylaxis is effective toward identifying which patients are the most likely to derive a net clinical benefit. Given the marked heterogeneity of cancer populations, future studies should incorporate validated risk stratification approaches [61] and evaluate individualized thromboprophylactic strategies rather than universal preventive regimens. Particular attention should be paid to contemporary anticoagulants, catheter type, tumour characteristics, evolving oncological therapies and contemporary central venous access practices, all of which may substantially influence both thrombotic and bleeding risk. Future research should focus on adequately powered randomized trials with standardized definitions of catheter-related thrombosis and systematic reporting of bleeding outcomes, particularly in the context of contemporary anticoagulants and modern oncological care. Furthermore, they should strictly minimize the heterogeneity of the patient population, for example, by focusing on a single cancer type, one type of central venous device, one anticoagulant strategy and ideally similar systemic anticancer treatments.

### Strengths and Limitations

The strengths of this review include a comprehensive literature search across multiple databases, prospective protocol registration in PROSPERO, and rigorous risk-of-bias assessment using validated tools. Adherence to the PRISMA methodology ensured the transparent reporting and systematic identification of relevant studies. In addition, the separate synthesis of randomized and non-randomized studies allowed for a more structured interpretation of the available evidence.

Several limitations should be acknowledged. First, the number of studies eligible for quantitative synthesis was limited, particularly for direct oral anticoagulants, which reduced the statistical power of pooled estimates. Second, the included studies differed with respect to patient populations, catheter types, anticoagulant regimens, and follow-up duration, which may contribute to clinical heterogeneity. Third, the definitions of catheter-related thrombosis varied across studies, ranging from symptomatic events to imaging-detected thrombosis, potentially affecting the comparability of reported outcomes. In addition, the relatively small number of events limited the precision of pooled estimates and reduced the ability to explore potential sources of heterogeneity through subgroup analyses. Another limitation is that the included studies spanned more than two decades, during which central venous access techniques, catheter materials and insertion practices evolved substantially. Consequently, older studies may not fully reflect current clinical practice, although their inclusion was consistent with the predefined eligibility criteria and allowed for a comprehensive synthesis of the available evidence. Finally, bleeding outcomes were inconsistently reported across studies, precluding formal quantitative synthesis and limiting the assessment of the net clinical benefit of prophylactic anticoagulation. These limitations are consistent with those reported in previous systematic reviews rather than the current evidence base and limitations unique to the present review. Therefore, the present findings should be interpreted as hypothesis-generating rather than definitive evidence supporting routine thromboprophylaxis in all patients with long-term central venous catheters.

## 5. Conclusions

Prophylactic anticoagulation in cancer patients with long-term central venous catheters remains a clinically relevant but unresolved issue. While some studies suggest a possible reduction in catheter-related thrombosis, the available evidence is limited by substantial heterogeneity, low certainty, and inconsistent bleeding reporting. Current data do not support universal thromboprophylaxis, although selected high-risk patients may potentially benefit from individualized preventive strategies. Further adequately powered randomized trials using standardized outcome definitions are needed to clarify the balance between thrombotic prevention and bleeding risk.

## Figures and Tables

**Table 1:** Characteristics of included studies.

Author, Year	Sample Size	Study Design	Population	CVC Type	Intervention	Comparator	Follow-Up	Outcomes Reported
Brandt, W. 2022 [21]	563	RCT	Newly diagnosed cancer or progression of known cancer after complete or partial remission and those who were initiating a new course of chemotherapy with a minimum treatment of 3 months	TIVAD, PICC	Prophylactic anticoagulation with apixaban 2,5 mg twice daily	Cancer patients receiving long-term CVC without prophylactic anticoagulation	210 days	Major venous thromboembolism, major bleeding
Young, A.M. 2009 [22]	1590	RCT	Histologically confirmed diagnosis of cancer, needed CVC insertion for administration of CHT, at least 16 years old, had adequate hepatic, renal, hematological functions	Indwelling CVC	Prophylactic anticoagulation with warfarin fixed or adjusted dose	Cancer patients receiving long-term CVC without prophylactic anticoagulation	45 months	CRT, major bleeding
Couban, S. 2005 [23]	255	Multicentre RCT	16 years of age or older with histologic or cytologic evidence of cancer who required an indwelling CVC for at least 7 days	Hickman type, PICC type, porta cath, PAS-port	Prophylactic anticoagulation with warfarin (1 mg daily)	Cancer patients receiving long-term CVC without prophylactic anticoagulation	3 months after CVC removal	CRT, bleeding
Lavau-Denes, S. 2013 [24]	407	RCT	(1) histological evidence of solid, invasive cancer or metastatic status; (2) presence of subclavian CVC inserted for less than 7 days; (3) starting the first line of chemotherapy; (4) aged 18 years or older; (5) life expectancy more than 3 months; (6) performance status between 0 and 2 (ambulatory); (7) platelets greater than 100 × 10^9^/L and normal APTT; (8) the capacity to provide informed consent	Long-term CVC	Prophylactic coagulation with warfarin 1 mg/daily and LMWH (isocoagulation dose)	Cancer patients receiving long-term CVC without prophylactic anticoagulation	3 months	CRT
Karthaus, M. 2006 [25]	439	RCT	Patients undergoing chemotherapy with documented cancer	Long-term CVC	Prophylactic anticoagulation with 5000 IU dalteparin	Cancer patients receiving long-term CVC without prophylactic anticoagulation	12 weeks	CRT, major bleeding
Cicco, M.D. 2009 [26]	450	RCT	Cancer patients, over 18 years of age, life expectancy at least 3 months, CVC administration for chemotherapy	TIVAD and tunnelled, exteriorized long-term CVC	Prophylactic anticoagulation with 1 mg/day acenocoumarin	Cancer patients receiving long-term CVC without prophylactic anticoagulation	2 months	CRT
Verso, M. 2005 [27]	385	RCT	18 years or older, scheduled for CVC insertion for chemotherapy, life expectancy over 3 months and adequate venous access to perform venography of the upper limb if the CVC was left to be in site for longer than 6 weeks	Long-term CVCs	Prophylactic anticoagulation with 40 mg enoxaparin	Cancer patients receiving long-term CVC without prophylactic anticoagulation	42 days (±2) days	CRT, major bleeding
Ikesaka, R. 2021 [28]	105	RCT	Adult (≥18 years) patients with active cancer who had a CVC inserted within 72 h of enrolment and had the capacity to provide written consent	PICC, port	Prophylactic anticoagulation with 10 mg rivaroxaban	Cancer patients receiving long-term CVC without prophylactic anticoagulation	90 (±3) days	CRT, major bleeding
Fagnani, D. 2006 [29]	1410	nRCT (observational prospective study)	Solid or hematological tumours, age over 18, CVD (port, indwelling CVC or PICC)	Port, indwelling CVC, PICC	Prophylactic anticoagulation with low-dose warfarin (1 mg/day)	Cancer patients receiving long-term CVC without prophylactic anticoagulation	3 years or until CVD removal	CRT
Boraks, P. 1998 [30]	223	nRCT	Hematological malignancies, adults	Indwelling CVCs	Prophylactic anticoagulation with low-dose warfarin (1 mg/day)	Cancer patients receiving long-term CVC without prophylactic anticoagulation	Max. 79 days	CRT
Lv, S. 2019 [31]	423	nRCT (observational prospective study)	≥18 years of age; diagnosis of gastric, lung, esophageal, breast, colorectal or ovarian cancer; scheduled for treatment via PICC insertion	PICC	Prophylactic anticoagulation with 10 mg rivaroxaban or 4000 IU enoxaparin	Cancer patients receiving long-term CVC without prophylactic anticoagulation	Until PICC removal (max 160 days)	CRT
Sang, D. 2025 [32]	314	nRCT (prospective cohort study)	Pathologically confirmed breast cancer; placement of PICC; age ≥18, 80≤ years old; normal function of major organs; Eastern Cooperative Oncology Group (ECOG) performance status of 0–2 and expected survival > 6 months; normal coagulation function before treatment	PICC	Prophylactic anticoagulation with 10 mg rivaroxaban	Cancer patients receiving long-term CVC without prophylactic anticoagulation	Until 6 months after catheterization	CRT
Tesselaar, M. 2005 [33]	243	nRCT	14–78 years of age, bone tumours, distal esophagus or stomach cancer, patients with distant metastases	Implantable pots (chest and arm)	Prophylactic anticoagulation with nadroparin or coumarin	Cancer patients receiving long-term CVC without prophylactic anticoagulation	1795 days	CRT, bleeding
Jia, B. 2025 [34]	822	NRCT (retrospective cohort study)	Adult cancer patients who underwent PICC or port implantation	PICC and port	Prophylactic anticoagulation with rivaroxaban 10 mg	Cancer patients receiving long-term CVC without prophylactic anticoagulation	Mean 293 days	CRT
Magagnoli, M. 2005 [35]	427	nRCT	Adult cancer patients with hematological and non-hematological malignancies	Vygon external devices, Groshong catheters, Port-a-cath	Prophylactic anticoagulation with low-dose warfarin (1 mg/day)	No clear control group	168 days	CRT, major bleeding
Magagnoli, M. 2006 [36]	228	nRCT (retrospective study)	Patients who underwent HDC treatments followed by autologous PBSCT but also breast cancer, sarcomas and other patients were included	Vygon external catheters, Groshong catheters	Prophylactic anticoagulation with low-dose warfarin (1 mg/day)	No clear control group	Mean 173 days	VRT, bleeding

**Table 2:** Summary of bleeding outcome definitions and reported major bleeding across included studies.

Author	Study Population	Definition	Major Bleeding Outcomes	Comments
Young, A.M. 2009 [22]	Histologically confirmed diagnosis of cancer, needed CVC insertion for administration of CHT, at least 16 years old, had adequate hepatic, renal, hematological functions	Intracranial, retroperitoneal, requiring transfusion or hospital admission or directly leading to death	<1% (no warfarin) vs. 2–3% (warfarin)	Author’s definition
Brandt, W. 2022 [21]	Newly diagnosed cancer or progression of known cancer after complete or partial remission and those who were initiating a new course of chemotherapy with a minimum treatment of 3 months	Major bleeding defined by the International Society on Thrombosis and Haemostasis as overt bleeding that was associated with a decrease in the hemoglobin level of 2 g/dL or more, led to transfusion of 2 or more units of packed red cells, occurred in a critical site, or contributed to death	6 patients (2.1%)	ISTH definition
Magagnoli, M. 2005 [35]	Adult cancer patients with hematological and non-hematological malignancies	Soft tissue bleeding requiring blood transfusion, hematemesis, hemoptysis, melena, macrohematuria, vaginal bleeding apart from that of normal menses epistaxis for more than one hour with gross blood loss, or retinal hemorrhages with impairment of vision	3.5% of patients	Author’s definition
Magagnoli, M. 2006 [36]	Patients who underwent HDC treatments followed by autologous PBSCT, but also breast cancer, sarcomas and other patients were included	No clear outcome definition (just “bleeding”)	4/228 patients (1.7%)	No formal definition
Couban, S. 2005 [23]	16 years of age or older with histologic or cytologic evidence of cancer who required an indwelling CVC for at least 7 days	Major bleeding was present if any of the following occurred: CNS bleeding, bleeding with hypotension (SBP < 80 mmHg or >30 mmHg decrease in SBP), bleeding associated with transfusion of more than 2 units of red cells in any 24 h period, or a decrease in hemoglobin by 20 g/L or more in any 24 h period	6 (2%) placebo vs. 0 (0%) warfarin	Study-specific definition (similar to ISTH criteria)
Karthaus, M. 2006 [25]	Patients undergoing chemotherapy with documented cancer	Major bleeding was defined as a fall in hemoglobin of 2 g/dL or more or a 6% or greater reduction in hematocrit with manifest hemorrhage; any intraocular, intraspinal or intracranial hemorrhage; bleeding requiring blood transfusion or fatal hemorrhage	17.5% (dalteparin) vs. 15% (placebo)	Study-specific definition (published before ISTH definition)
Ikesaka, R. 2021 [28]	Adult (≥18 years) patients with active cancer who had a CVC inserted within 72 h of enrolment and had the capacity to provide written consent	Major bleeding defined by the International Society on Thrombosis and Haemostasis as overt bleeding that was associated with a decrease in the hemoglobin level of 2 g/dL or more, led to transfusion of 2 or more units of packed red cells, occurred in a critical site, or contributed to death.	1.9% (rivaroxaban) vs. 0 (standard care)	ISTH definition

**Table 3:** Summary of findings (GRADE).

Synthesis	Outcome	No. of Participants (Studies)	Relative Effect	Certainty of Evidence (GRADE)
LMWH	CRT	1022 (3)	0.77 (0.53, 1.12)	Low
Rivaroxaban	CRT	593 (2)	0.23 (0.11, 0.48)	Very low

## Data Availability

No new data were created or analyzed in this study. Data sharing is not applicable to this article.

## Supplementary Materials

The following supporting information can be downloaded at https://www.mdpi.com/article/10.3390/jcm15145566/s1, Supplementary Table S1: PRISMA 2020 checklist; Supplementary Table S2: Studies excluded from systematic review with reason for exclusion (includes cited references: [37,38,39,40,41,42,43,44,45,46]); Supplementary Table S3: Studies excluded from meta-analysis with reason for exclusion; Figures S1 and S2: ROB2; Figures S3 and S4: ROBINS-I; File S1: Database: Clinical Trials; File S2: Database: PubMed; File S3: Database: Scopus; File S4: Database: WebofScience.

## Abbreviations

The following abbreviations are used in this manuscript:

CRT	catheter-related thrombosis
CVD	central venous device
CVC	central venous catheter
PICC	peripherally inserted central catheter
TIVAD	totally implantable venous access device
CI	confidence interval
RR	risk ratio
LMWH	low-molecular-weight heparin
DOAC	direct oral anticoagulant
RCT	randomized controlled trial
CRVT	catheter-related venous thrombosis
ASH	America Society of Haematology
ITAC	Initiative on Thrombosis and Cancer
ASCO	American Society of Clinical Oncology
ISTH	International Society on Thrombosis and Haemostasis
