# Supplementary material for: A Systematic Review and Meta-Analysis of Prophylactic Anticoagulation for the Prevention of Catheter-Related Thrombosis in Adult Cancer Patients with Long-Term Central Venous Catheters: Current Evidence, Clinical Uncertainties and Future Directions
Source: J Clin Med. 2026 Jul 15;15(14):5566. doi: 10.3390/jcm15145566 (PMC13413132; doi:10.3390/jcm15145566)
Supplement: Supplementary file 1 [file jcm-15-05566-s001.zip › jcm-4380838-supplementary/Supplementary materials/File S1 Database Clinical Trials.pdf]

Date: 07.12.2025

Database: Clinical Trials

„String": Central Venous Catheter thrombosis/Catheter related thrombosis/ anticoagulants

No studies found: 5
