# Supplementary material for: A Systematic Review and Meta-Analysis of Prophylactic Anticoagulation for the Prevention of Catheter-Related Thrombosis in Adult Cancer Patients with Long-Term Central Venous Catheters: Current Evidence, Clinical Uncertainties and Future Directions
Source: J Clin Med. 2026 Jul 15;15(14):5566. doi: 10.3390/jcm15145566 (PMC13413132; doi:10.3390/jcm15145566)
Supplement: Supplementary file 1 [file jcm-15-05566-s001.zip › jcm-4380838-supplementary/Supplementary materials/File S2 Database PubMed.pdf]

Database: Pubmed

Date: 30.11.2025

Search strategy: **(cancer[tiab] OR cancers[tiab] OR oncolog*[tiab] OR "Neoplasms"[Mesh]) AND (CVC[tiab] OR "central venous catheter"[tiab] OR "central line"[tiab] OR TIVAD[tiab] OR "totally implantable venous access device"[tiab] OR portacath[tiab] OR "porta-cath"[tiab] OR PICC[tiab]) AND (anticoagul*[tiab] OR prophylaxis[tiab] OR thromboprophylaxis[tiab]) AND ("catheter-related thrombosis"[tiab] OR CRT[tiab] OR CRVT[tiab] OR "central line thrombosis"[tiab] OR thrombosis[tiab] OR thrombotic[tiab] OR "major bleeding"[tiab] OR bleeding[tiab] OR hemorrhag*[tiab])** Filters: **Clinical Study, Clinical Trial, Guideline, Observational Study, Preprint, Randomized Controlled Trial**

1. No of records: 34
2. No of records after duplicates removal:
3. No of Records screened:

   → records excluded (with reason):

4. No of Full- length articles assessed:
   → no of full- length excluded (with reason):
5. Studies included in qualitative synthesis:
6. Studies included in quantitative synthesis:
