# Supplementary material for: A Systematic Review and Meta-Analysis of Prophylactic Anticoagulation for the Prevention of Catheter-Related Thrombosis in Adult Cancer Patients with Long-Term Central Venous Catheters: Current Evidence, Clinical Uncertainties and Future Directions
Source: J Clin Med. 2026 Jul 15;15(14):5566. doi: 10.3390/jcm15145566 (PMC13413132; doi:10.3390/jcm15145566)
Supplement: Supplementary file 1 [file jcm-15-05566-s001.zip › jcm-4380838-supplementary/Supplementary materials/File S3 Database Scopus.pdf]

Database: Scopus

Date: 07.12.2025

Search strategy: **TITLE-ABS-KEY (cancer OR cancers OR oncolog* OR neoplasm*) AND TITLE-ABS-KEY (CVC OR "central venous catheter" OR "central line" OR TIVAD OR "totally implantable venous access device" OR "venous access device*" OR "central venous access device*" OR portacath OR "porta-cath" OR PICC OR "implantable port*") AND TITLE-ABS-KEY (anticoagul* OR prophylaxis OR thromboprophylaxis) AND TITLE-ABS-KEY ("catheter-related thrombosis" OR CRT OR CRVT OR "central line thrombosis" OR thrombosis OR thrombotic OR "major bleeding" OR bleeding OR hemorrhag*) AND TITLE-ABS-KEY (adult*) AND (LIMIT-TO (DOCTYPE , "ar"))**

1. No of records: 324
2. No of records after duplicates removal:
3. No of Records screened:

   → records excluded (with reason):

4. No of Full- length articles assessed:
   → no of full- length excluded (with reason):
5. Studies included in qualitative synthesis:
6. Studies included in quantitative synthesis:
