# Supplementary material for: A Systematic Review and Meta-Analysis of Prophylactic Anticoagulation for the Prevention of Catheter-Related Thrombosis in Adult Cancer Patients with Long-Term Central Venous Catheters: Current Evidence, Clinical Uncertainties and Future Directions
Source: J Clin Med. 2026 Jul 15;15(14):5566. doi: 10.3390/jcm15145566 (PMC13413132; doi:10.3390/jcm15145566)
Supplement: Supplementary file 1 [file jcm-15-05566-s001.zip › jcm-4380838-supplementary/Supplementary materials/File S4 Database WebofScience.pdf]

Database: Web Of Science

Date: 07.12.2025

Search strategy: TS=(cancer OR cancers OR oncolog* OR neoplasm*)

AND

TS=(

CVC OR "central venous catheter" OR "central line"

OR TIVAD OR "totally implantable venous access device"

OR "venous access device*" OR "central venous access device*"

OR portacath OR "porta-cath" OR PICC OR "implantable port*"

)

AND

TS=(

anticoagul* OR prophylaxis OR thromboprophylaxis

)

AND

TS=(

"catheter-related thrombosis" OR CRT OR CRVT

OR "central line thrombosis" OR thrombosis OR thrombotic

OR "major bleeding" OR bleeding OR hemorrhag*

)

AND

TS=(adult OR adults)

Filter: Articles, Clinical Trials

1. No of records: 215
2. No of records after duplicates removal:
3. No of Records screened:

   → records excluded (with reason):

4. No of Full- length articles assessed:
   → no of full- length excluded (with reason):

5. Studies included in qualitative synthesis:
6. Studies included in quantitative synthesis:
