# Supplementary material for: A Systematic Review and Meta-Analysis of Prophylactic Anticoagulation for the Prevention of Catheter-Related Thrombosis in Adult Cancer Patients with Long-Term Central Venous Catheters: Current Evidence, Clinical Uncertainties and Future Directions
Source: J Clin Med. 2026 Jul 15;15(14):5566. doi: 10.3390/jcm15145566 (PMC13413132; doi:10.3390/jcm15145566)
Supplement: Supplementary file 1 [file jcm-15-05566-s001.zip › jcm-4380838-supplementary/Supplementary materials/Table S2 Studies excluded from systematic review with reason for exclusion.pdf]

Supplementary Table S2. Studies excluded from systematic review with reason for exclusion.

Author, year	Reason for exclusion from systematic review
Palumbo, A. 2008 [37]	Wrong study design
Niers, T.M.H. 2007 [38]	Wrong population (short-term catheters)
Monreal, M. 1996 [39]	Could not be retrieved
Laporte, S. 2004 [40]	Wrong study design
Haddad, R.A. 2018 [41]	Wrong population
Calderero Aragon, V. [42]	Language of the full- text
Abdelkefi, A. 2004 [43]	Wrong population
Heaton, D.C. 2002 [44]	Could not be retrieved
Ratcliffe, M. 1999 [45]	Could not be retrieved
Paauw, J.D. 2008 [46]	Could not be retrieved

37. Palumbo, A.; Rajkumar, S.V.; Dimopoulos, M.A.; Richardson, P.G.; San Miguel, J.; Barlogie, B.; Harousseau, J.; Zonder, J.A.; Cavo, M.; Zangari, M.; et al. Prevention of thalidomide- and lenalidomide-associated thrombosis in myeloma. *Leukemia* **2008**, *22*, 414–423. https://doi.org/10.1038/sj.leu.2405062. PMID: 18094721.
38. Niers, T.M.; Di Nisio, M.; Klerk, C.P.; Baarslag, H.J.; Büller, H.R.; Biemond, B.J. Prevention of catheter-related venous thrombosis with nadroparin in patients receiving chemotherapy for hematologic malignancies: A randomized, placebo-controlled study. *J. Thromb. Haemost.* **2007**, *5*, 1878–1882. https://doi.org/10.1111/j.1538-7836.2007.02660.x. PMID: 17723127.
39. Monreal, M.; Alastrue, A.; Rull, M.; Mira, X.; Muxart, J.; Rosell, R.; Abad, A. Upper extremity deep venous thrombosis in cancer patients with venous access devices--prophylaxis with a low molecular weight heparin (Fragmin). *Thromb. Haemost.* **1996**, *75*, 251–253. PMID: 8815570.
40. Laporte, S.; Decousus, H.; Mismetti, P. Thrombosis prophylaxis in cancer patients with a central venous catheter. *Arch. Intern. Med.* **2004**, *164*, 459; https://doi.org/10.1001/archinte.164.4.459-a. PMID: 14980999.
41. Haddad, R.A.; Alnimer, Y.; Abdalla, A.; Ríos-Bedoya, C.F.; Bachuwa, G. Is Peripherally Inserted Central Catheter-Related Thrombosis Associated with ABO Blood Group? A Case-Control Pilot Study. *Clin. Appl. Thromb. Haemost.* **2018**, *24*, 1297–1300. https://doi.org/10.1177/1076029618770289. PMID: 29683035; PMCID: PMC6714765.
42. Calderero Aragón, V.; de Gregorio Ariza, M.A.; Pazo Cid, R.; Puértolas Hernández, T.; Lostalé Latorre, F.; Artal Cortés, A.; Antón Torres, A. Role of low molecular weight heparins in prophylaxis of thromboembolic events on oncological patients with indwelling central venous catheter. *Med. Clin.* **2009**, *133*, 365–370. (In Spanish). https://doi.org/10.1016/j.medcli.2009.05.015. PMID: 19646715.
43. Abdelkefi, A.; Ben Othman, T.; Kammoun, L.; Chelli, M.; Romdhane, N.B.; Kriaa, A.; Ladeb, S.; Torjman, L.; Lakhal, A.; Achour, W.; et al. Prevention of central venous line-related thrombosis by continuous infusion of low-dose unfractionated heparin, in patients with haemato-oncological disease. A randomized controlled trial. *Thromb. Haemost.* **2004**, *92*, 654–661. https://doi.org/10.1160/TH04-02-0087. PMID: 15351864.

44. Heaton, D.C.; Han, D.Y.; Inder, A. Minidose (1 mg) warfarin as prophylaxis for central vein catheter thrombosis. *Intern. Med. J.* **2002**, *32*, 84–88. PMID: 11885848.
45. Ratcliffe, M.; Broadfoot, C.; Davidson, M.; Kelly, K.F.; Greaves, M. Thrombosis, markers of thrombotic risk, indwelling central venous catheters and antithrombotic prophylaxis using low-dose warfarin in subjects with malignant disease. *Clin. Lab. Haematol.* **1999**, *21*, 353–357. https://doi.org/10.1046/j.1365-2257.1999.00250.x. PMID: 10646078.
46. Paauw, J.D.; Borders, H.; Ingalls, N.; Boomstra, S.; Lambke, S.; Fedeson, B.; Goldsmith, A.; Davis, A.T. The incidence of PICC line-associated thrombosis with and without the use of prophylactic anticoagulants. *JPEN J. Parenter. Enteral Nutr.* **2008**, *32*, 443–447. https://doi.org/10.1177/0148607108319801. PMID: 18596317.
