# Supplementary material for: A Systematic Review and Meta-Analysis of Prophylactic Anticoagulation for the Prevention of Catheter-Related Thrombosis in Adult Cancer Patients with Long-Term Central Venous Catheters: Current Evidence, Clinical Uncertainties and Future Directions
Source: J Clin Med. 2026 Jul 15;15(14):5566. doi: 10.3390/jcm15145566 (PMC13413132; doi:10.3390/jcm15145566)
Supplement: Supplementary file 1 [file jcm-15-05566-s001.zip › jcm-4380838-supplementary/Supplementary materials/Table S3 Studies excluded from meta-analysis with reason for exclusion.pdf]

Supplementary Table S3. Studies excluded from meta-analysis with reason for exclusion.

Author, year	Reason for exclusion from meta-analysis
Fagnani, D. 2006 [29]	Insufficient reporting of outcome data required for effect estimation
Cicco, M.D 2009 [26]	Insufficient reporting of outcome data required for effect estimation
Boraks, P. 1998 [30]	The study could not be pooled with any other included study due to the absence of a comparable comparator or outcome definition
Ikesaka, R. 2021 [28]	The study could not be pooled with any other included study due to the absence of a comparable comparator or outcome definition
Brandt, W. [21]	Insufficient reporting of outcome data required for effect estimation
Young, A.M. 2009 [22]	The study population did not meet the predefined age eligibility criteria
Couban, S. 2005 [23]	The study population did not meet the predefined age eligibility criteria
Tesselaar, M. 2004 [33]	The study population did not meet the predefined age eligibility criteria
Jia, B. 2025 [34]	Insufficient reporting of outcome data required for effect estimation
Magagnoli, M. 2005 [35]	The study lacked a control group
Magagnoli, M. 2006 [36]	The study lacked a control group

21. Brandt, W.; Brown, C.; Wang, T.F.; Tagalakis, V.; Shivakumar, S.; Ciuffini, L.A.; Mallick, R.; Wells, P.S.; Carrier, M. Efficacy and safety of apixaban for primary prevention of thromboembolism in patients with cancer and a central venous catheter: A subgroup analysis of the AVERT Trial. *Thromb. Res.* **2022**, *216*, 8–10. https://doi.org/10.1016/j.thromres.2022.05.014. PMID: 35660801.
22. Young, A.M.; Billingham, L.J.; Begum, G.; Kerr, D.J.; Hughes, A.I.; Rea, D.W.; Shepherd, S.; Stanley, A.; Sweeney, A.; Wilde, J.; Wheatley, K.; WARP Collaborative Group, UK. Warfarin thromboprophylaxis in cancer patients with central venous catheters (WARP): An open-label randomised trial. *Lancet* **2009**, *373*, 567–574. https://doi.org/10.1016/S0140-6736(09)60205-1. PMID: 19217991.
23. Couban, S.; Goodyear, M.; Burnell, M.; Dolan, S.; Wasi, P.; Barnes, D.; Macleod, D.; Burton, E.; Andreou, P.; Anderson, D.R. Randomized placebo-controlled study of low-dose warfarin for the prevention of central venous catheter-associated thrombosis in patients with cancer. *J. Clin. Oncol.* **2005**, *23*, 4063–4069. https://doi.org/10.1200/JCO.2005.10.192. PMID: 15767639.
26. De Cicco, M.; Matovic, M.; Balestreri, L.; Steffan, A.; Pacenzia, R.; Malafronte, M.; Fantin, D.; Bertuzzi, C.A.; Fabiani, F.; Morassut, S.; et al. Early and short-term acenocumarine or dalteparin for the prevention of central vein catheter-related thrombosis in cancer patients: A randomized controlled study based on serial venographies. *Ann. Oncol.* **2009**, *20*, 1936–1942. https://doi.org/10.1093/annonc/mdp235. PMID: 19567452.
28. Ikesaka, R.; Siegal, D.; Mallick, R.; Wang, T.F.; Witham, D.; Webb, C.; Carrier, M.; Canadian Venous Thromboembolism Research Network (CanVECTOR). Thromboprophylaxis with rivaroxaban in patients with malignancy and central venous lines (TRIM-Line): A two-center open-label pilot randomized controlled trial. *Res. Pract. Thromb. Haemost.* 2021, 5, e12517. https://doi.org/10.1002/rth2.12517. PMID: 34027291; PMCID: PMC8116834.
29. Fagnani, D.; Franchi, R.; Porta, C.; Pugliese, P.; Borgonovo, K.; Bertolini, A.; Duro, M.; Ardizzoia, A.; Filipazzi, V.; Isa, L.; et al. Thrombosis-related complications and mortality in cancer patients with central venous devices: An observational study on the effect of antithrombotic prophylaxis. *Ann. Oncol.* **2007**, *18*, 551–555. https://doi.org/10.1093/annonc/mdl431. PMID: 17158773.

30. Boraks, P.; Seale, J.; Price, J.; Bass, G.; Ethell, M.; Keeling, D.; Mahendra, P.; Baglin, T.; Marcus, R. Prevention of central venous catheter associated thrombosis using minidose warfarin in patients with haematological malignancies. *Br. J. Haematol.* **1998**, *101*, 483–486. https://doi.org/10.1046/j.1365-2141.1998.00732.x. PMID: 9633891.
33. Tesselaar, M.E.; Ouwerkerk, J.; Nooy, M.A.; Rosendaal, F.R.; Osanto, S. Risk factors for catheter-related thrombosis in cancer patients. *Eur. J. Cancer* **2004**, *40*, 2253–2259. https://doi.org/10.1016/j.ejca.2004.06.023. PMID: 15454250.
34. Jia, B.; Lv, H.; Gong, T.; Wang, X.; Ma, Q.; Ren, Y.; Zhang, L.; Zhong, D. Evaluating Rivaroxaban for Thromboprophylaxis in Cancer Patients with Long-Term Venous Access Devices: A Retrospective Cohort Study. *Vasc. Health Risk Manag.* **2025**, *21*, 917–925. https://doi.org/10.2147/VHRM.S550388. PMID: 41215801; PMCID: PMC12596838.
35. Magagnoli, M.; Masci, G.; Castagna, L.; Zucali, P.A.; Morenghi, E.; Pedicini, V.; Santoro, A. Prophylaxis of central venous catheter-related thrombosis with minidose warfarin: Analysis of its use in 427 cancer patients. *Anticancer Res.* **2005**, *25*, 3143–3147. PMID: 16080578.
36. Magagnoli, M.; Masci, G.; Castagna, L.; Pedicini, V.; Poretti, D.; Morenghi, E.; Brambilla, G.; Santoro, A. Prophylaxis of central venous catheter-related thrombosis with minidose warfarin in patients treated with high-dose chemotherapy and peripheral-blood stem-cell transplantation: Retrospective analysis of 228 cancer patients. *Am. J. Hematol.* **2006**, *81*, 1–4. https://doi.org/10.1002/ajh.20512. PMID: 16369969.
