# Supplementary figures and images for: A Systematic Review and Meta-Analysis of Prophylactic Anticoagulation for the Prevention of Catheter-Related Thrombosis in Adult Cancer Patients with Long-Term Central Venous Catheters: Current Evidence, Clinical Uncertainties and Future Directions
Source: J Clin Med. 2026 Jul 15;15(14):5566. doi: 10.3390/jcm15145566 (PMC13413132; doi:10.3390/jcm15145566)

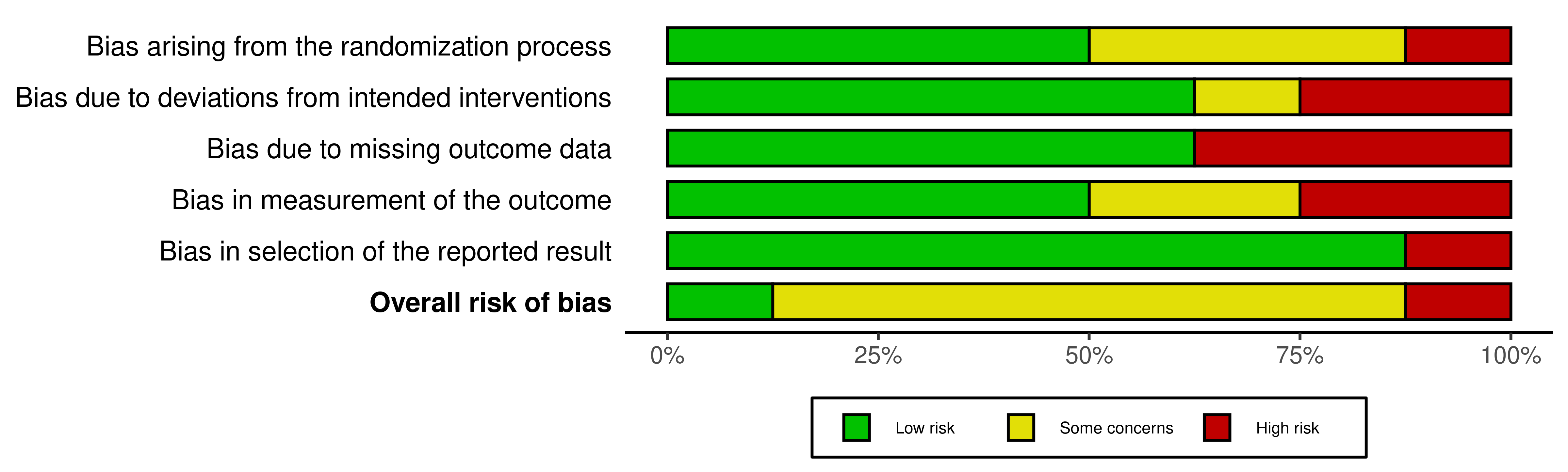

Supplement: Supplementary file 1 [file jcm-15-05566-s001.zip › jcm-4380838-supplementary/Supplementary materials/Figure S1ROB2.png]

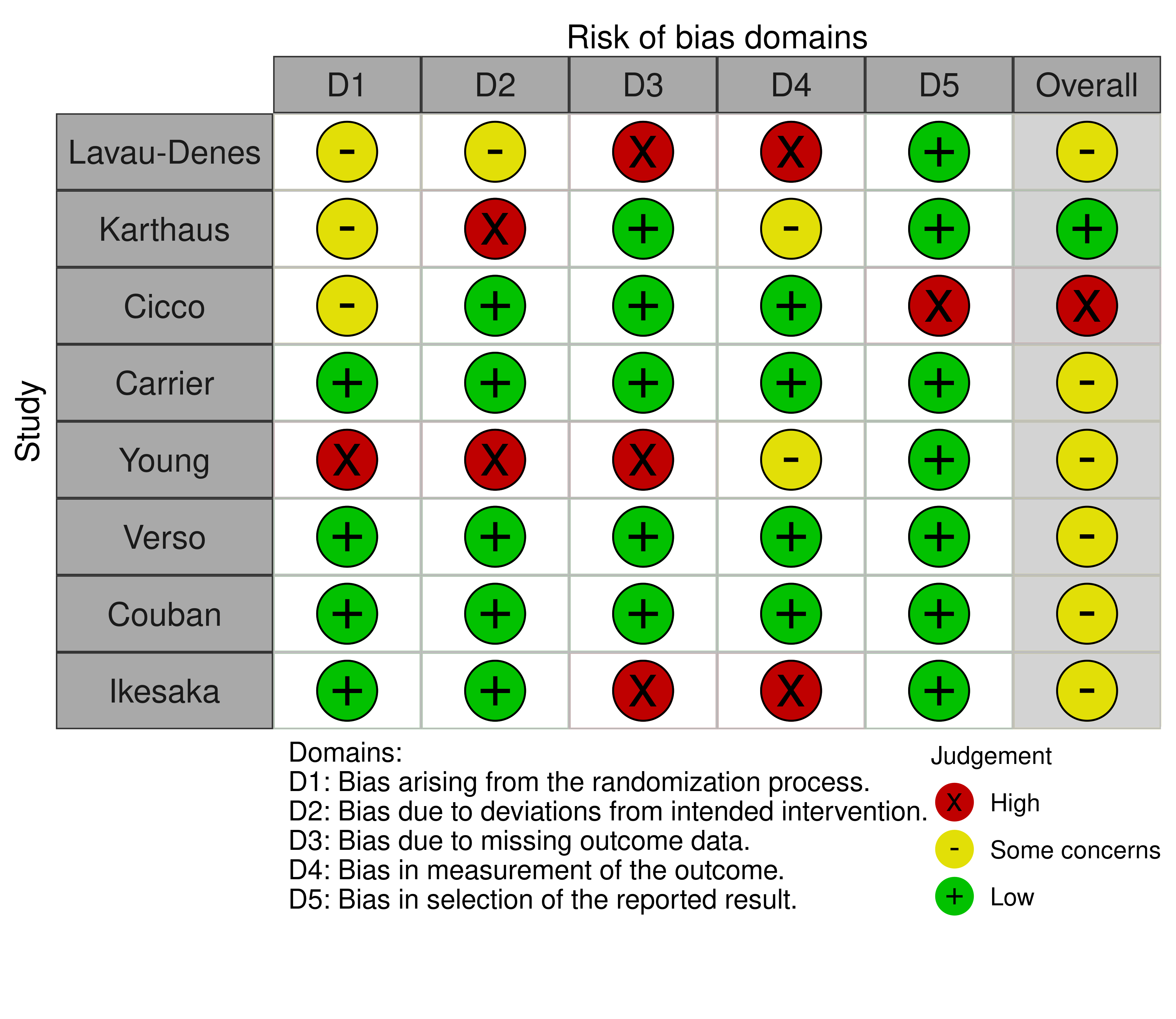

Supplement: Supplementary file 1 [file jcm-15-05566-s001.zip › jcm-4380838-supplementary/Supplementary materials/Figure S2ROB2.png]

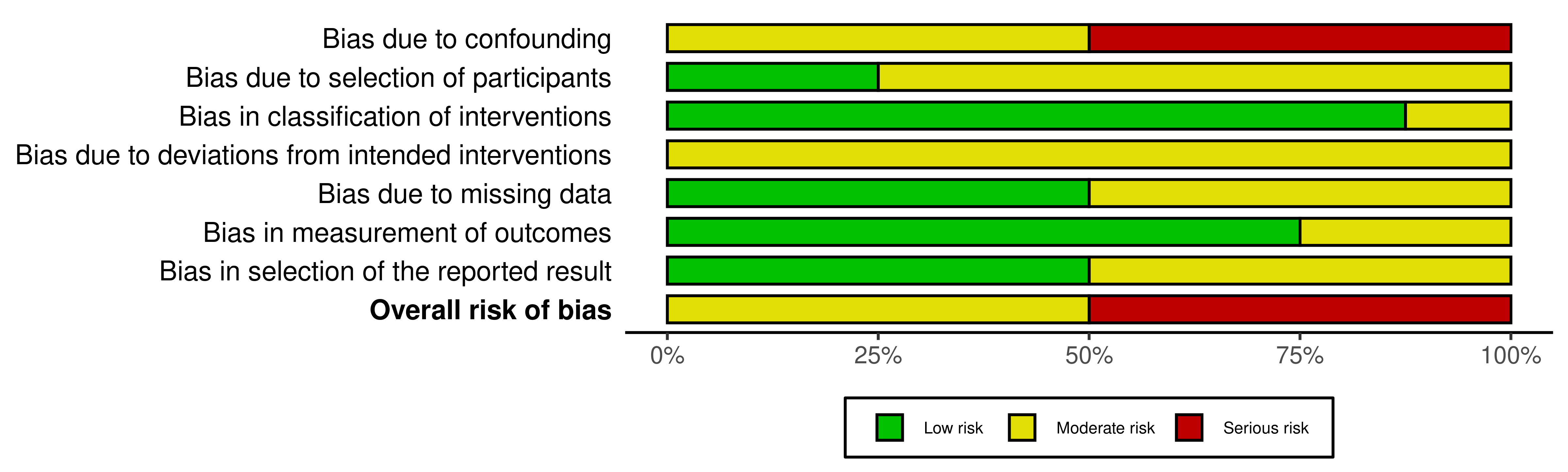

Supplement: Supplementary file 1 [file jcm-15-05566-s001.zip › jcm-4380838-supplementary/Supplementary materials/Figure S3ROBINS-I.png]

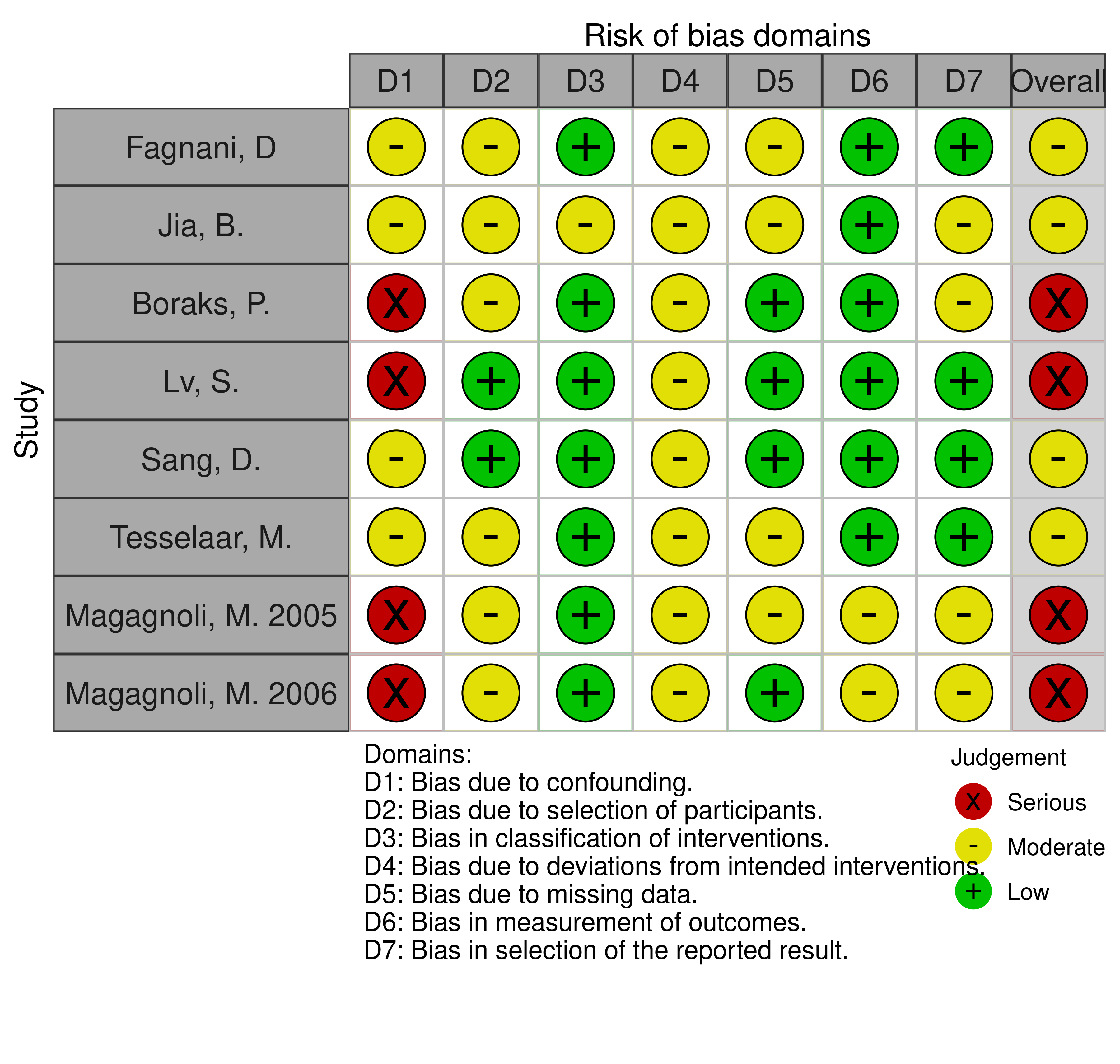

Supplement: Supplementary file 1 [file jcm-15-05566-s001.zip › jcm-4380838-supplementary/Supplementary materials/Figure S4ROBINS-I.png]
